# Supplementary material for: Coumarin-Based Triapine Derivatives and Their Copper(II) Complexes: Synthesis, Cytotoxicity and mR2 RNR Inhibition Activity
Source: Biomolecules. 2021 Jun 9;11(6):862. doi: 10.3390/biom11060862 (PMC8230303; doi:10.3390/biom11060862)
Supplement: Supplementary file 1 [file biomolecules-11-00862-s001.zip › biomolecules-1227758-supplementary.pdf]

Electronic Supplementary Materials

for

Coumarin-based triapine derivatives and their copper(II) complexes: synthesis, cytotoxicity and mR2 RNR inhibition activity

Iryna Stepanenko [1]*, Maria V. Babak [2], Gabriella Spengler [3], Marta Hammerstad [4], Ana Popović-Bijelić [5], Sergiu Shova [6], Gabriel E. Büchel [7], Denisa Darvasiová [8], Peter Rapta [8] and Vladimir B. Arion [1]*

[1]Institute of Inorganic Chemistry, University of Vienna, Währinger Strasse 42, A-1090 Vienna, Austria; iryna.stepanenko@univie.ac.at (I.S.); vladimir.arion@univie.ac.at (V.B.A.)

[2]Drug Discovery Lab, Department of Chemistry, City University of Hong Kong, 83 Tat Chee Avenue, Hong Kong SAR, China; mbabak@cityu.edu.hk (M.V.B.)

[3]Department of Medical Microbiology, Albert Szent-Györgyi Health Center and Faculty of Medicine, University of Szeged, Semmelweis utca 6, H-6725 Szeged, Hungary; spengler.gabriella@med.u-szeged.hu (G.S.)

[4]Section for Biochemistry and Molecular Biology, Department of Biosciences, University of Oslo, P.O. Box 1066, Blindern, NO-0316 Oslo, Norway; marta.hammerstad@ibv.uio.no (M.H.)

[5]Faculty of Physical Chemistry, University of Belgrade, Studentski trg 12-16, 11158 Belgrade, Serbia; ana@ffh.bg.ac.rs (A.P.-B.)

[6]"Petru Poni" Institute of Macromolecular Chemistry, Aleea Gr. Ghica Voda 41A, 700487 Iasi, Romania; shova@icmpp.ro (S.S.)

[7]ChemConsult GmbH, PO Box 43, 9485 Nendeln, Liechtenstein; gabriel.buechel@gmail.com (G.E.B.)

[8]Institute of Physical Chemistry and Chemical Physics, Faculty of Chemical and Food Technology, Slovak University of Technology in Bratislava, Radlinského 9, SK-812 37 Bratislava, Slovakia; denisa.darvasiova@stuba.sk (D.D.); peter.rapta@stuba.sk (P.R.)

*corresponding author: iryna.stepanenko@univie.ac.at (I.S.)

*corresponding author: vladimir.arion@univie.ac.at (V.B.A.)

Contents:

Fig. S1. ^{1}H,^{1}H-NOESY spectrum of $\mathbf{D_1}$ in DMSO-d_6 S3

Fig. S2. ^{1}H,^{1}H-NOESY spectrum of $\mathbf{D_2}$ in DMSO-d_6 S3

Fig. S3. Emission and absorption spectra of $\mathbf{HL^1}$-$\mathbf{HL^3}$ and $\mathbf{H_2L^4}$ S4

Fig. S4. The cyclic voltammograms of (a) **1** (black trace) and **4** (green trace) in cathodic part, and (b) **HL**^{3} in anodic part **S6**
Fig. S5. Concentration-effect curves of **HL**^{1}**-HL**^{3} and **H**$_2$**L**^{4} and their respective Cu(II) complexes in MDA-MB-231 cells upon 72 h exposure **S7**
Scheme S1. Synthesis of 4-chloromethyl-2-dimethoxymethylpyridine, reagents and conditions **S7**
Scheme S2. Synthesis of 7-hydroxy-3-(piperazine-1-carbonyl)-2*H*-chromen-2-one (**H**) **S8**
Synthesis of building blocks E and H **S8**
Scheme S3. Protection of aldehyde group of **C**$_1$ and atom labeling scheme used in the NMR resonances assignment of **D**$_1$ **S14**
Scheme S4. Protection of aldehyde group of **C**$_2$ and atom labeling scheme used in the NMR resonances assignment of **D**$_2$ **S15**
Scheme S5. Atom labeling schemes used in the NMR resonances assignment of **H, I**$_1$ and **I**$_2$ **S15**
Scheme S6. Atom labeling schemes used in the NMR resonances assignment of **J**$_1$**, J**$_{h1}$ and **J**$_2$ **S16**
Scheme S7. Hydrolysis of two acetal groups of **I**$_2$ in HCl solution **S17**
Scheme S8. Last steps in the synthesis of TSCs **S17**
Scheme S9. Atom labeling schemes used in the NMR resonances assignment of **HL**^{1}**–HL**^{3} **S19**
Scheme S10. Atom labeling scheme used in the NMR resonances assignment of **H**$_2$**L**^{4} **S19**
Table S1. Optimisation of reaction conditions in step (x) to improve the yield of **I**$_1$ **S20**
References **S20**

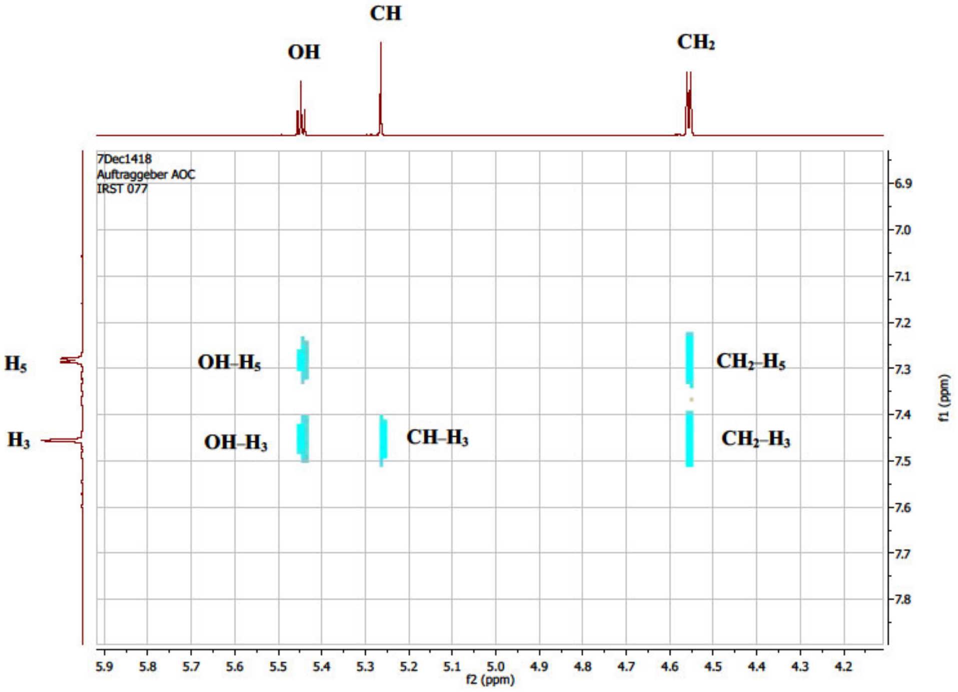

Fig. S1. ^{1}H,^{1}H-NOESY spectrum of $\mathbf{D_1}$ in DMSO-d_6.

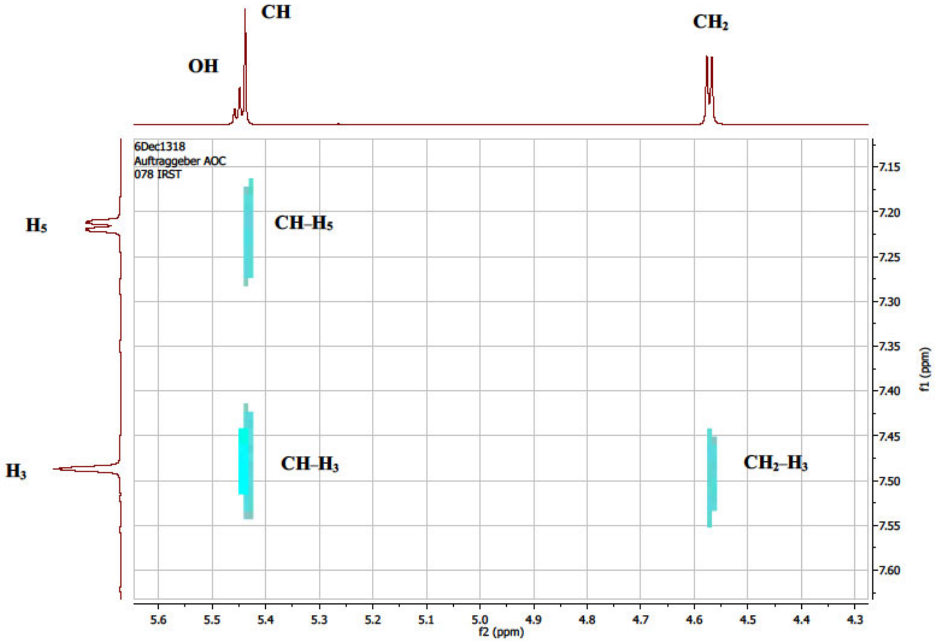

Fig. S2. ^{1}H,^{1}H-NOESY spectrum of $\mathbf{D_2}$ in DMSO-d_6.

H_2O

HL[1]

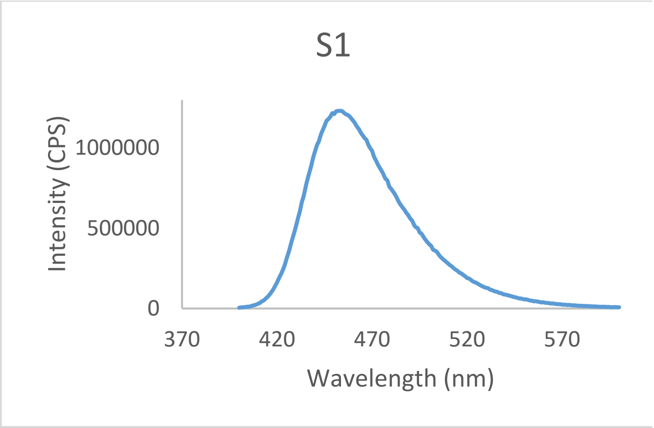

λ_{em} = 453 nm

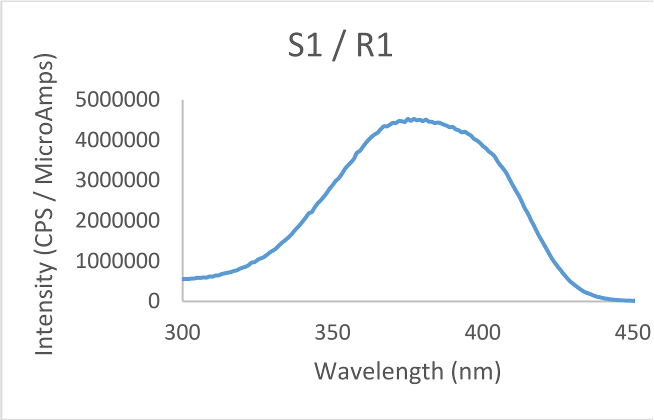

λ_{ex} = 377 nm

HL[2]

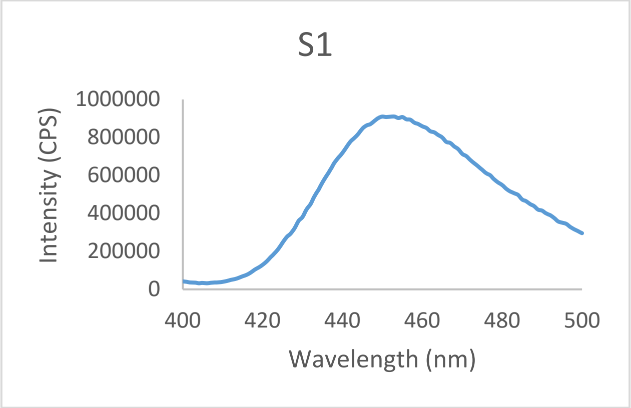

λ_{em} = 453 nm

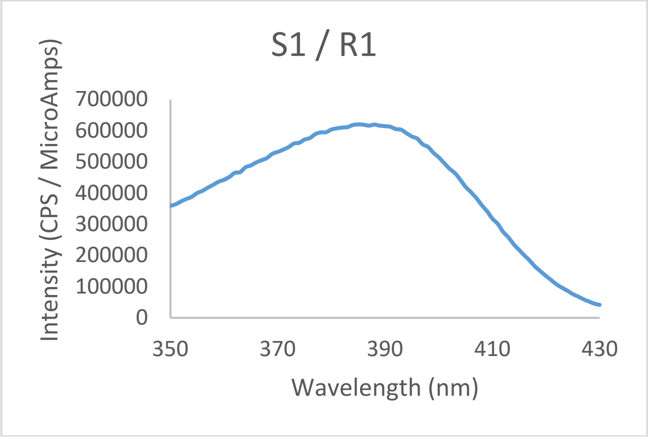

λ_{ex} = 388 nm

HL[3]

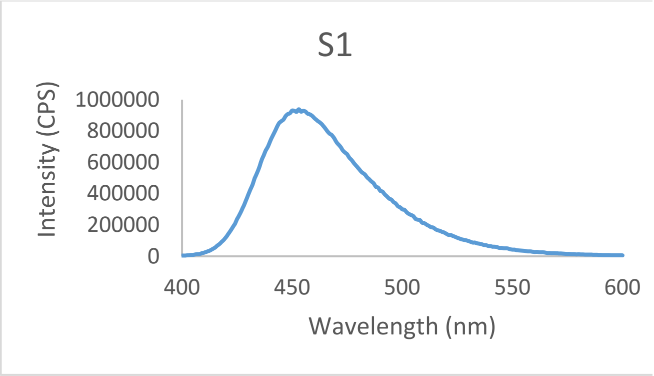

λ_{em} = 453 nm

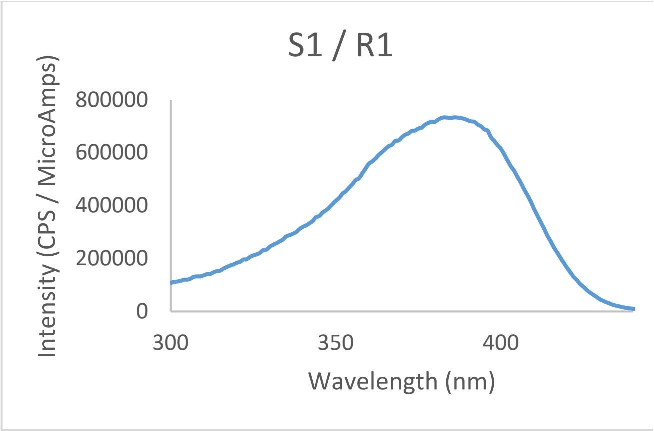

λ_{ex} = 386 nm

1% DMSO/H_2O

HL[1]

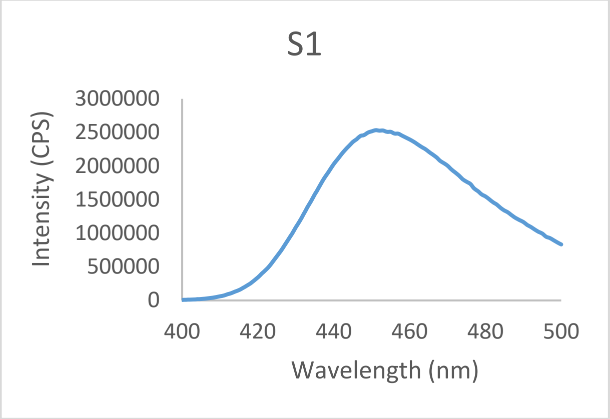

λ_{em} = 451 nm

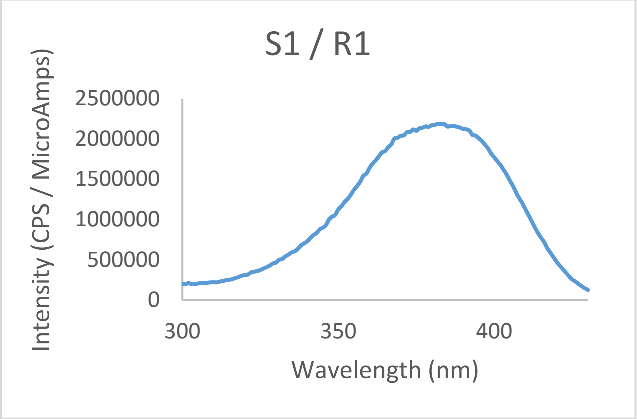

λ_{ex} = 382 nm

HL[2]

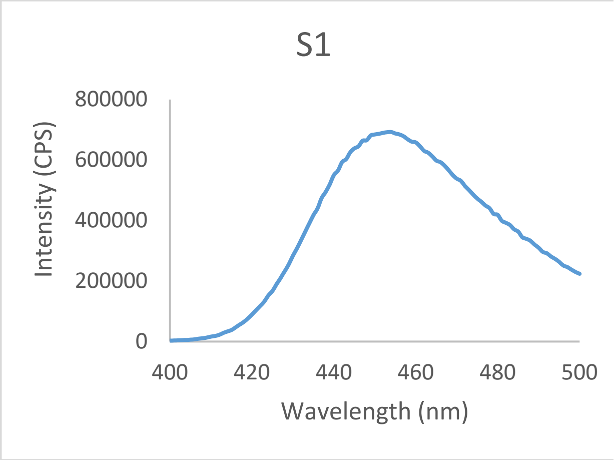

λ_{em} = 454 nm

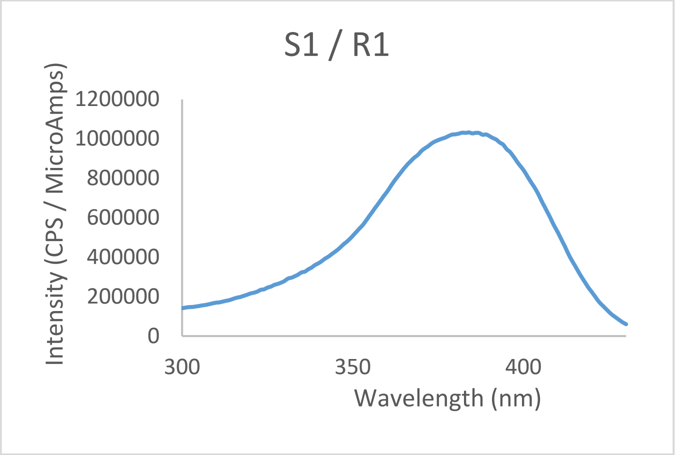

λ_{ex} = 384 nm

HL[3]

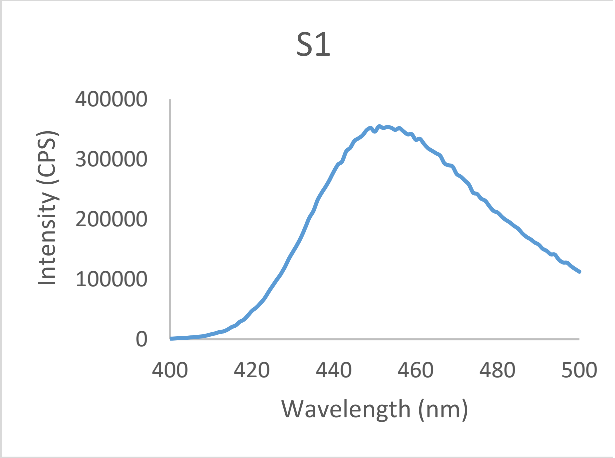

λ_{em} = 451 nm

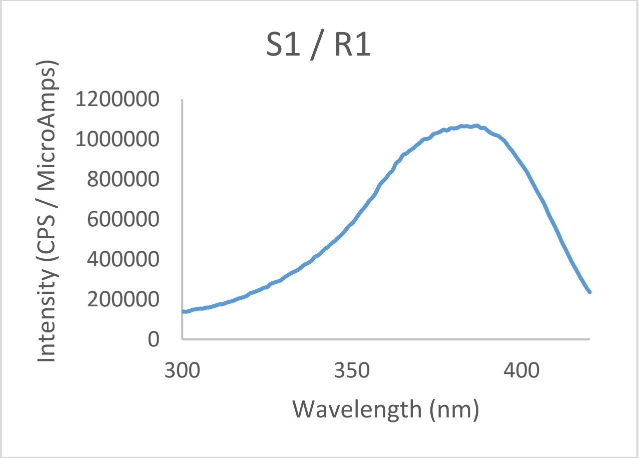

λ_{ex} = 387 nm

H_2L^4

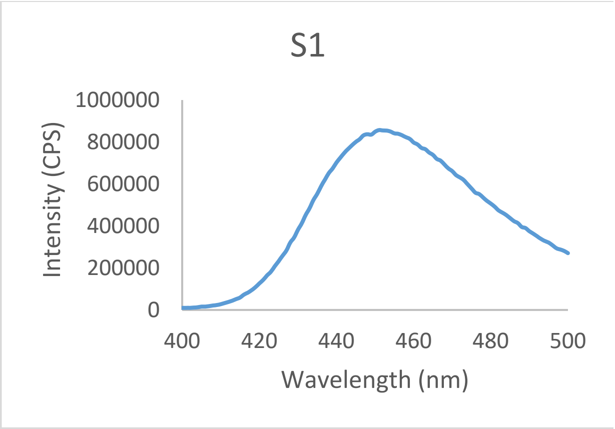

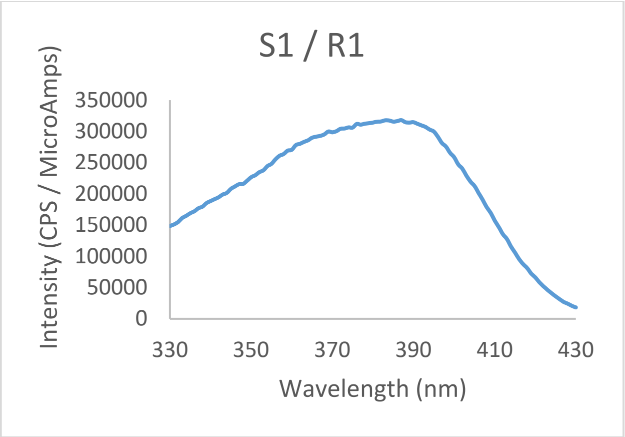

λ_{em} = 451 nm

λ_{ex} = 387 nm

Fig. S3. Emission and absorption spectra of **HL^1–HL^3** and **H_2L^4**.

(a)

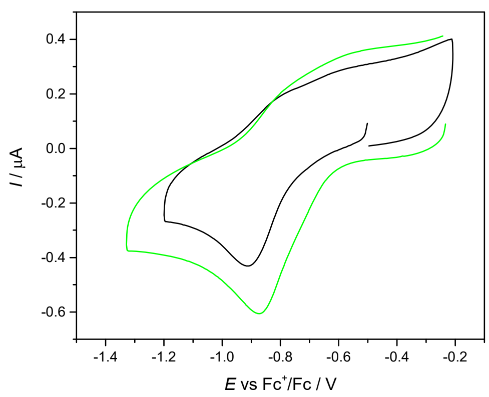

(b)

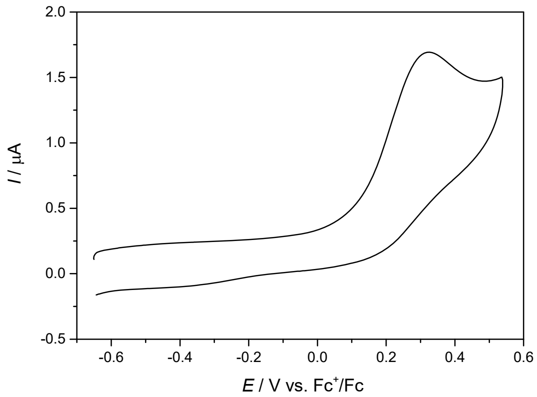

Fig. S4. The cyclic voltammograms of (a) **1** (black trace) and **4** (green trace) in cathodic part, and (b) **HL^3** in anodic part measured in DMSO/nBu$_4$NPF$_6$ at glassy-carbon working electrode, at scan rate of 100 mV s^{-1}.

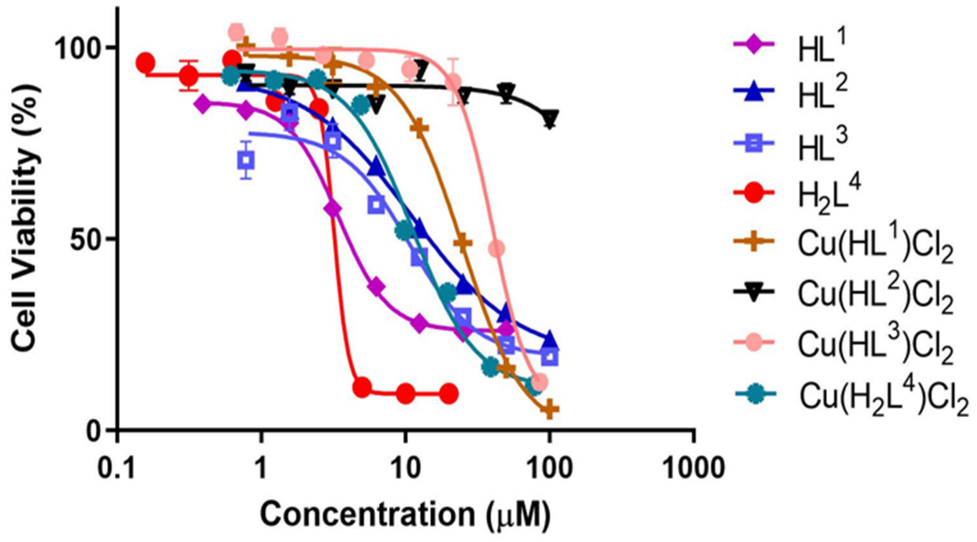

Fig. S5. Concentration-effect curves of **HL¹–HL³** and **H₂L⁴** and their respective Cu(II) complexes in MDA-MB-231 cells upon 72 h exposure.

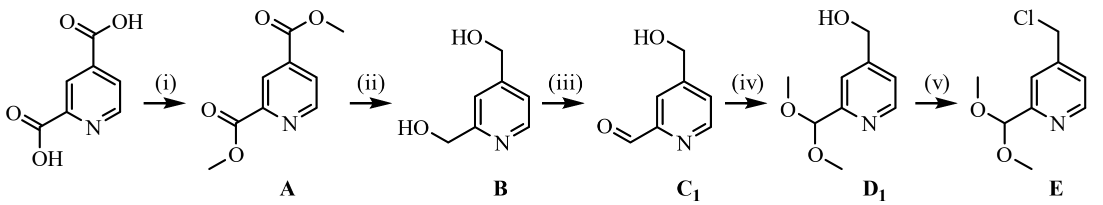

Scheme S1. Synthesis of 4-chloromethyl-2-dimethoxymethylpyridine. Reagents and conditions: (i) $SOCl_2$, MeOH, reflux, overnight; (ii) $NaBH_4$, C_2H_5OH, 0 °C for 1 h, room temperature for 3 h, overnight under reflux, purification by column chromatography; (iii) SeO_2, dioxane/water, under argon, 100 °C for 3 h, purification by column chromatography; (iv) trimethyl orthoformate, methanesulfonic acid, dry MeOH, under argon, 78 °C for 48 h; (v) $SOCl_2$, dry CH_2Cl_2, –80 °C.

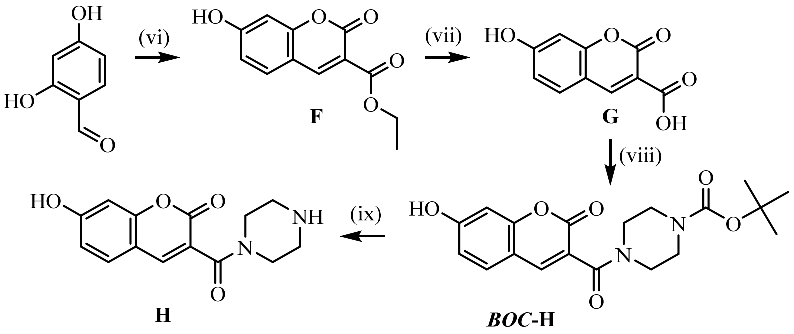

Scheme S2. Synthesis of 7-hydroxy-3-(piperazine-1-carbonyl)-2*H*-chromen-2-one (**H**). Reagents and conditions: (vi) diethyl malonate, piperidine, C_2H_5OH, reflux, 1 h; (vii) 0.5 M NaOH, room temperature, 24 h, 1M HCl, 1 h; (viii) *t*-butyl piperazine-1-carboxylate, EDCl, HOBt, DIEA, dry DMF, room temperature, 12 h; (ix) CF_3COOH, CH_2Cl_2, room temperature, 2 h, purification by column chromatography.

Synthesis of building blocks E and H

*Synthesis of 4-chloromethyl-2-dimethoxymethylpyridine (**E**)*. Dimethyl pyridine-2,4-dicarboxylate **A** (Scheme S1) was obtained in the first step by the esterification of 2,4-pyridinedicarboxylic acid in the presence of thionyl chloride in MeOH in good yield (76.5%) as reported in the literature.[1] The formation of **A** was confirmed by the presence of the positive ion ESI-MS peaks at *m*/*z* 196.01 and 217.98 attributed to $[M+H]^+$ and $[M+Na]^+$, respectively. In the next step ester **A** was reduced to 2,4-pyridinedimethanol **B** in moderate yield (31–39%) by using $NaBH_4$ in THF at room temperature as reported previously for 2,6-pyridinedimethanol.[2] Column chromatography of the crude product on SiO_2 by using ethanol as eluent showed the presence of three other fractions with R_f = 0.87, 0.5 and 0.3 in addition to species **B** with R_f = 0.75. Reduction of **A** with $NaBH_4$ in boiling ethanol, as reported previously for 2,5-pyridinedimethanol,[3] afforded diol **B** in very good yield (75.7%). Only one side product (R_f = 0.5) was present in the reaction mixture and the diol **B** could be easily purified by column chromatography (SiO_2, C_2H_5OH, fr_1 (R_f = 0.75)). The positive ion ESI mass spectrum of **B** showed peaks at *m*/*z* 140.01 and 161.98, which were assigned to $[M+H]^+$ and $[M+Na]^+$, respectively. The ^{1}H NMR spectrum of **B** in DMSO-d_6 showed signals

attributed to three doublets of three C*H* pyridine ring protons, two triplets of two hydroxyl groups and overlapped doublets of CH_2 groups. The most laborious step was the oxidation of the hydroxyl group in position 2 of the pyridine ring in **B**. The reaction was performed in the presence of SeO_2 in dioxane/water 38:1 mixture under argon at 100 °C for 3 h as reported previously for 2-formyl-5-hydroxymethylpyridine.[4] However, the conversion was not selective and resulted in several products. Two monooxidised species, the required 2-formyl-4-hydroxymethylpyridine (**C_1**) and 4-formyl-2-hydroxymethylpyridine **C_2**, were isolated in approximately the same yields (20–25%) as yellow oils. In addition, 2,4-diformylpyridine **$C_{1,2}$** was obtained (17–23% yield) along with unreacted diol **B**. The products were separated by column chromatography on SiO_2 (CH_2Cl_2/MeOH 8:1, R_{f1} (**$C_{1,2}$**) = 0.57, R_{f2} (**C_1**) = 0.48, R_{f3} (**C_2**) = 0.45, R_{f4} (**B**) = 0.3). The **C_1** and **C_2** gave in the negative ion ESI mass spectra peaks at *m*/*z* 135.9 attributed to $[M–H]^-$. The ^1H NMR spectra of **C_1** and **C_2** revealed a set of one C*H* signal of aldehyde group, three C*H* signals originated from C*H* pyridine protons, one triplet of hydroxyl group and one doublet of methylene group protons. The main difference in the ^1H NMR spectra between the two isomers is the downfield shift of C*H* signals of the aldehyde group in **C_2** relative to that for **C_1** by 0.12 ppm. The identification of two monooxidised products as **C_1** and **C_2** was possible by inspection of 2D NMR spectra of the protected products, **D_1** and **D_2**, obtained from **C_1** and **C_2** in the next step (see Scheme S3 and Scheme S4, Fig. S1 and Fig. S2). The protection was performed with trimethyl orthoformate in the presence of methanesulfonic acid as catalyst in dry methanol as described quite recently for 2-formyl-5-hydroxymethylpyridine.[5] The quantitative yields were obtained in both cases, **D_1** and **D_2**, but double excess of catalyst for **D_2** was required. Both oily products **D_1** and **D_2** were used in the next step without any additional purification. Pure products can be obtained by column chromatography (SiO_2, CH_2Cl_2/MeOH 10:1 or 6:1). They showed in ESI mass spectra peaks attributed to $[M+H]^+$, $[M+Na]^+$ and $[M–H]^-$ ions. The recorded ^1H NMR resonances of three C*H* pyridine protons, hydroxyl group, methylene group and CH_3 of acetal group protons in **D_1** and **D_2** were quite similar. The only remarkable ^1H NMR shift difference should be mentioned for the C*H* proton of the acetal group which is seen at 5.26 ppm in **D_1** and 5.44 ppm in **D_2**. In addition, the chemical shifts of ^{13}C NMR resonances in **D_1** and **D_2** are quite different. The identification of **D_1** and **D_2** was done by using 2D NMR spectra. According to ^1H,^1H-NOESY spectra for (2-(dimethoxymethyl)pyridin-4-yl)methanol **D_1**, the OH and two methylene group protons have two couplings with H3 and H5, whereas the C*H* proton of the acetal group – only with H3 (Scheme

S3, Fig. S1). In the case of 4-(dimethoxymethyl)pyridin-2-yl)methanol $\mathbf{D_2}$, protons of methylene groups have coupling with H3, whereas the C*H* proton of the acetal group – with H3 and H5 as shown in Fig. S2 (see also Scheme S4). Finally the chlorination of $\mathbf{D_1}$ was conducted with thionyl chloride in dry CH_2Cl_2 at –80°C as reported previously for 5-chloromethyl-2-dimethoxymethyl-pyridine[3] to give pure building block **E** in excellent yield (94.1%).

Synthesis of 7-hydroxy-3-(piperazine-1-carbonyl)-2H-chromen-2-one (**H**). The ethyl 7-hydroxycoumarin-3-carboxylate **F** and 7-hydroxycoumarin-3-carboxylic acid **G** (Scheme S2) were prepared according to the literature,[6] but the basic hydrolysis to produce **G** was optimised to reach better reproducibility. In the next step by coupling the species **G** with *BOC*-protected piperazine in the presence of peptide coupling reagents (EDCl, HOBt) in dry DMF the protected amide *BOC*-**H** was obtained as reported previously.[7] *BOC*-deprotection of the latter was performed in the presence CF_3COOH in CH_2Cl_2 to yield the final intermediate as **H**·TFA adduct.

Experimental part

Pyridine-2,4-dicarboxylic acid dimethyl ester (**A** in Scheme S1).[1] To 2,4-pyridinedicarboxylic acid (10.3 g, 0.062 mol) suspended in MeOH (40 mL) and cooled in an ice bath was added dropwise thionyl chloride (12 mL, 0.165 mol). The mixture was stirred under reflux overnight. Then MeOH was removed under reduced pressure and the residue was partitioned between the saturated aqueous $NaHCO_3$ (75 mL) and CH_2Cl_2 (2 × 75 mL). The organic phase was separated, washed with water (3 × 75 mL) and dried over Na_2SO_4. The solvent was removed under reduced pressure to give the product as a white solid. Yield: 9.2 g, 76.5%. Positive ion ESI-MS (ACN/MeOH+1% H_2O): *m/z* 196.01 $[M+H]^+$, 217.98 $[M+Na]^+$.

2,4-Pyridinedimethanol (**B** in Scheme S1). Dimethyl-2,4-pyridinecarboxylate **A** (5.03 g, 0.03 mol) was suspended in ethanol (70 mL) and the mixture was cooled to 0 °C in an ice bath. Sodium borohydride (4.53 g, 0.12 mol) was added to the mixture in small portions. After the reaction mixture was stirred at 0 °C for 1 h, the ice bath was removed. The reaction mixture was further stirred at room temperature for 3 h, and then heated under reflux overnight. The solvent was removed under reduced pressure, and the residue was dissolved in acetone (70 mL). Saturated aqueous K_2CO_3 (70 mL) was added, and the mixture was heated at reflux for 1 h. The yellow layer was decanted from the reaction mixture and evaporated. The product was purified

chromatographically on silica by using C_2H_5OH as eluent. The first fraction (R_{f1} = 0.75) was collected as a light yellow oil and dried in high vacuum to give a white solid. Yield: 2.7 g, 75.7%. Positive ion ESI-MS (ACN/MeOH+1% H_2O): *m/z* 140.01 $[M+H]^+$, 161.98 $[M+Na]^+$. ^1H NMR (500 MHz, DMSO-d_6) δ, ppm: 8.39 (d, *J* = 5.0 Hz, 1H, pyC*H*), 7.44 (d, *J* = 0.7 Hz, 1H, pyC*H*), 7.16 (d, *J* = 5.0 Hz, 1H, pyC*H*), 5.44 (t, *J* = 5.7 Hz, 1H, CH_2OH), 5.40 (t, *J* = 5.8 Hz, 1H, CH_2OH), 4.55 (d, *J* = 5.8 Hz, 4H, CH_2OH).

2-Formyl-4-hydroxymethylpyridine (**C_1** in Scheme S1). The diol **B** (3.92 g, 0.028 mol) was dissolved in dioxane (30 mL) at 40–60 °C, then water (0.78 mL) and SeO_2 (1.56 g, 0.014 mol) were added. The mixture was heated under argon at 100 °C for 3 h. The reaction mixture was filtered through Celite and washed with dioxane (15 mL). The filtrate and washings were evaporated and the residue was purified chromatographically on silica by using CH_2Cl_2/MeOH 8:1 as eluent. The second fraction (R_{f2} = 0.48) was collected to give the product **C_1** as a light yellow oil, which was dried in high vacuum to give a beige solid. Yield: 1.0 g, 25.9%. Positive ion ESI-MS for **C_1** (ACN/MeOH+1% H_2O): *m/z* 160.28 $[M+Na]^+$; negative: *m/z* 135.97 $[M–H]^–$. ^1H NMR (**C_1**, 500 MHz, DMSO-d_6) δ, ppm: 10.00 (s, 1H, CO*H*), 8.75 (d, *J* = 5.0 Hz, 1H, pyC*H*), 7.90 (dd, *J* = 1.6, 0.8 Hz, 1H, pyC*H*), 7.67 – 7.60 (m, 1H, pyC*H*), 5.62 (t, *J* = 5.6 Hz, 1H, CH_2OH), 4.65 (d, *J* = 4.9 Hz, 2H, CH_2OH). Two additional products were also isolated: *4-formyl-2-hydroxymethylpyridine* (**C_2**, SiO_2, CH_2Cl_2/MeOH 8:1, R_{f3} = 0.45, yield 18–21%) and *2,4-diformylpyridine* (**$C_{1,2}$**, SiO_2, CH_2Cl_2/MeOH 8:1, R_{f1} = 0.57, yield 17–23%). Negative ion ESI-MS for **C_2** (ACN/MeOH+1% H_2O): *m/z* 135.93 $[M–H]^–$. ^1H NMR (**C_2**, 500 MHz, DMSO-d_6) δ, ppm: 10.12 (s, 1H, CO*H*), 8.78 (d, *J* = 4.9 Hz, 1H, pyC*H*), 7.92 – 7.84 (m, 1H, pyC*H*), 7.68 (dd, *J* = 5.3, 1.1 Hz, 1H, pyC*H*), 5.66 (t, *J* = 5.2 Hz, 1H, CH_2OH), 4.67 (d, *J* = 4.3 Hz, 2H, CH_2OH). Negative ion ESI-MS for **$C_{1,2}$** (ACN/MeOH+1% H_2O): *m/z* 134.02 $[M–H]^–$. ^1H NMR (**$C_{1,2}$**, 500 MHz, DMSO-d_6) δ, ppm: 10.19 (s, 1H, CO*H*), 10.08 (s, 1H, CO*H*), 9.11 (d, *J* = 4.8 Hz, 1H, pyC*H*), 8.34 – 8.27 (m, 1H, pyC*H*), 8.11 (dd, *J* = 4.8, 1.6 Hz, 1H, pyC*H*).

2-Dimethoxymethyl-4-hydroxymethylpyridine (**D_1** in Scheme S1). 2-Formyl-4-hydroxymethylpyridine **C_1** (1.0 g, 7.29 mmol), trimethyl orthoformate (3.16 mL, 28.9 mmol), methanesulfonic acid (74.0 µL, 1.14 mmol) and absolute methanol (14 mL) were combined under argon in a Schlenk tube. The reaction mixture was stirred at 78 °C for 48 h. After cooling down to room temperature the solvent was evaporated under reduced pressure. The residue was dissolved in $CHCl_3$ (50 mL) and washed with saturated aqueous K_2CO_3 (25 mL) and brine (25 mL). The

aqueous phase was extracted with $CHCl_3$ (3 × 50 mL). The combined organic layers were dried over $MgSO_4$ and the solvent was removed under reduced pressure to give a green oil of crude product, which was used without further purification in the next step. Yield: 1.19 g, 87.8%. The product can be purified chromatographically on silica by using CH_2Cl_2/MeOH 10:1 as eluent and collecting the fraction with R_f = 0.43 to produce an yellow oil. Positive ion ESI-MS for $\mathbf{D_1}$ (ACN/MeOH+1% H_2O): *m/z* 184.23 $[M+H]^+$, 206.23 $[M+Na]^+$; negative: *m/z* 182.05 $[M–H]^–$. ^1H NMR ($\mathbf{D_1}$, 700 MHz, DMSO-d_6) δ, ppm: 8.46 (d, J = 5.0 Hz, 1H, H_6), 7.46 (s, 1H, H_3), 7.28 (d, J = 5.7 Hz, 1H, H_5), 5.45 (t, J = 5.8 Hz, 1H, CH_2OH), 5.26 (s, 1H, $HC(OCH_3)_2$), 4.56 (d, J = 5.8 Hz, 2H, CH_2OH), 3.29 (s, 6H, $HC(OCH_3)_2$). ^{13}C NMR ($\mathbf{D_1}$, 176 MHz, DMSO-d_6) δ, ppm: 156.83 (C_2), 152.25 (C_4), 148.39 (C_6), 120.90 (C_5), 117.89 (C_3), 104.05 ($HC(OCH_3)_2$), 61.51 (CH_2OH), 53.37 ($HC(OCH_3)_2$). The synthesis of $\mathbf{D_2}$ was performed under the same reaction conditions starting from $\mathbf{C_2}$, but with a double excess of catalyst. Yield: 75–81%. Positive ion ESI-MS for $\mathbf{D_2}$ (ACN/MeOH+1% H_2O): *m/z* 184.27 $[M+H]^+$, 206.28 $[M+Na]^+$; negative: *m/z* 182.03 $[M–H]^–$. ^1H NMR ($\mathbf{D_2}$, 600 MHz, DMSO-d_6) δ, ppm: 8.48 (d, J = 5.4 Hz, 1H, H_6), 7.49 (s, 1H, H_3), 7.22 (d, J = 5.0 Hz, 1H, H_5), 5.45 (t, 1H, CH_2OH), 5.44 (s, 1H, $HC(OCH_3)_2$), 4.57 (d, J = 5.6 Hz, 2H, CH_2OH), 3.27 (s, 6H, $HC(OCH_3)_2$). ^{13}C NMR ($\mathbf{D_2}$, 151 MHz, DMSO-d_6) δ, ppm: 162.30 (C_2), 148.63 (C_6), 146.90 (C_4), 119.60 (C_5), 117.68 (C_3), 101.25 ($HC(OCH_3)_2$), 64.12 (CH_2OH), 52.70 ($HC(OCH_3)_2$). For atom labeling used in assignment of resonances in NMR spectra of $\mathbf{D_1}$ and $\mathbf{D_2}$ see Scheme S3 and Scheme S4, respectively.

4-Chloromethyl-2-dimethoxymethylpyridine (**E** in Scheme S1). 2-Dimethoxymethyl-4-hydroxymethylpyridine $\mathbf{D_1}$ (0.23 g, 1.27 mmol) was dissolved in dry CH_2Cl_2 (20 mL) and cooled to –80 °C. Thionyl chloride (110 µL, 1.5 mmol) was added dropwise. The reaction mixture was stirred overnight, while it was allowed to slowly reach the room temperature. Next day the reaction mixture was cooled to –80 °C and triethylamine (230 µL, 1.65 mmol) was added dropwise. The content in the flask was allowed to reach the room temperature and stirred for additional 1 h. Then saturated aqueous $NaHCO_3$ (10 mL) and brine (10 mL) were added and the resulting mixture was extracted with CH_2Cl_2 (3 × 50 mL). The combined organic layers were dried over $MgSO_4$ and the solvent was removed under reduced pressure to give a red-brown oil. Yield: 0.24 g, 94.1%. Positive ion ESI-MS (ACN/MeOH+1% H_2O): *m/z* 202.43 $[M+H]^+$, 224.39 $[M+Na]^+$. ^1H NMR (600 MHz, DMSO-d_6) δ, ppm: 8.56 (d, J = 5.0 Hz, 1H, H_6), 7.55 (s, 1H, H_3), 7.43 (dd, J = 5.0, 1.7 Hz, 1H, H_5), 5.30 (s, 1H, CH), 4.83 (s, 2H, CH_2), 3.31 (s, 6H, CH_3). ^{13}C NMR (151 MHz, DMSO-d_6) δ,

ppm: 157.56 (C_2), 149.17 (C_6), 146.82 (C_4), 123.08 (C_5), 120.12 (C_3), 103.71 (*C*H), 53.47 (*C*H_3), 44.03 (*C*H_2).

Ethyl 7-hydroxy-2-oxo-2H-chromene-3-carboxylate (**F** in Scheme S2).[6] 2,4-Dihydroxybenzaldehyde (1.51 g, 10.9 mmol), diethyl malonate (2.47 mL, 16.3 mmol) and piperidine (0.8 mL, 8.15 mmol) were dissolved in ethanol (15 mL) and refluxed for 1 h. Then the reaction mixture was stirred at room temperature for 24 h and evaporated to dryness. The yellow powder of crude **F** was obtained by adding ethyl acetate (10 mL) and hexane (100 mL) and short treatment in ultrasound bath. The product was filtered off, dried in vacuo and used without further purification in the next step. Yield: 2.5 g, almost quantitative. Positive ion ESI-MS (ACN/MeOH+1% H_2O): *m/z* 235.06 $[M+H]^+$, 257.04 $[M+Na]^+$; negative: *m/z* 232.83 $[M–H]^–$. ^1H NMR (500 MHz, DMSO-d_6) δ, ppm: 8.46 (s, 1H, coumC*H*), 7.51 (d, *J* = 8.8 Hz, 1H, coumC*H*), 6.52 (dd, *J* = 8.7, 2.0 Hz, 1H, coumC*H*), 6.31 (d, *J* = 1.8 Hz, 1H, coumC*H*), 4.21 (q, *J* = 7.1 Hz, 2H, CH_2CH_3), 1.27 (t, *J* = 7.1 Hz, 3H, CH_2CH_3).

7-Hydroxycoumarin-3-carboxylic acid (**G** in Scheme S2). Ethyl ester of 7-hydroxycoumarin-3-carboxylic acid **F** (1.77 g, 7.6 mmol) was dissolved in 0.5 M aqueous solution of NaOH (40 mL) and the solution was left to stir at room temperature for 24 h. Solvent was removed from the reaction mixture by ½ of the original volume and then 1 M HCl (25 mL) was added. The suspension was stirred for 1 h and cooled to 4 °C. The beige precipitate was filtered off, washed with cold water (5–7 mL) and dried in vacuo. Yield: 1.1 g, 71.0%. Positive ion ESI-MS (ACN/MeOH+1% H_2O): *m/z* 206.97 $[M+H]^+$, 228.97 $[M+Na]^+$; negative: *m/z* 204.78 $[M–H]^–$. ^1H NMR (500 MHz, DMSO-d_6) δ, ppm: 12.88 (s, 1H, COO*H*), 11.06 (s, 1H, O*H*), 8.70 (s, 1H, coumC*H*), 7.76 (d, *J* = 8.6 Hz, 1H, coumC*H*), 6.85 (dd, *J* = 8.6, 2.2 Hz, 1H, coumC*H*), 6.75 (d, *J* = 2.2 Hz, 1H, coumC*H*).

7-Hydroxy-3-(piperazine-1-carbonyl)-2H-chromen-2-one (**H** in Scheme S2). Step (viii): A mixture of 7-hydroxycoumarin-3-carboxylic acid **G** (1.00 g, 5 mmol), *t*-butyl piperazine-1-carboxylate (0.93 g, 5 mmol), EDCl (1.34 g, 7.5 mmol), HOBt (1.7 g, 12.5 mmol) and DIEA (0.5 mL) in dry DMF (2.5 mL) was stirred at room temperature for 12 h.[7] Then water (5 mL) was added. The crude *BOC*-intermediate (*BOC*-**H**) was extracted with CH_2Cl_2, the organic phase was washed with water, dried over Na_2SO_4 and solvent removed under reduced pressure. Step (ix): CF_3COOH (TFA, 3 mL) was added dropwise to the *BOC*-intermediate (*BOC*-**H**) in CH_2Cl_2 (15 mL) and stirred at room temperature for 2 h. The wine-red oily residue obtained after solvent

evaporation was dried in vacuo at 60 °C, then mixed with CH_2Cl_2/MeOH 3:1 (15 mL) and stirred for ca. 30 min. The beige precipitate of **H**·TFA was filtered off and dried (0.63 g). Additionally, some amount of **H**·TFA was obtained from the filtrate by column chromatography on SiO_2 using CH_2Cl_2/MeOH 3:1 as eluent (fr_3, 0.15 g). Total yield of **H**·TFA: 0.78 g, 41.0%. Anal. Calcd for **H**·TFA, %: TFA, 29.36. Found, %: TFA, 28.76. Positive ion ESI-MS (ACN/MeOH+1% H_2O): *m/z* 275.07 $[M+H]^+$, 297.06 $[M+Na]^+$; negative: *m/z* 272.93 $[M–H]^-$. ^1H NMR (**H**·TFA, 500 MHz, DMSO-d_6) δ, ppm: 10.89 (brs, 1H, O*H* or N*H*), 8.84 (brs, 2H, O*H* or N*H*), 8.15 (s, 1H, coumC*H*), 7.63 (d, *J* = 8.6 Hz, 1H, coumC*H*), 6.86 (dd, *J* = 8.5, 2.3 Hz, 1H, coumC*H*), 6.78 (d, *J* = 2.2 Hz, 1H, coumC*H*), 3.79 (brs, 2H, pipCH_2), 3.58 (brs, 2H, pipCH_2), 3.17 (brs, 2H, pipCH_2), 3.10 (brs, 2H, pipCH_2). TFA free 7-hydroxy-3-(piperazine-1-carbonyl)-2H-chromen-2-one **H** as yellow solid was isolated after treatment with Et_3N as a base. The same procedure was used in situ in the next step (see step (x)). ^1H NMR (**H**, 700 MHz, DMSO-d_6) δ, ppm: 8.13 (s, 1H, H_4), 7.61 (d, *J* = 8.6 Hz, 1H, H_5), 6.86 (dd, *J* = 8.5, 2.0 Hz, 1H, H_6), 6.78 (s, 1H, H_8), 3.77 (brs, 2H, pipCH_2), 3.56 (brs, 2H, pipCH_2), 3.08 (brs, 2H, pipCH_2), 3.02 (brs, 2H, pipCH_2). ^{13}C NMR (**H**, 176 MHz, DMSO-d_6) δ, ppm: 163.60 (C_2 or C_{11}), 162.46 (C_7), 158.06 (C_2 or C_{11}), 155.73 (C_9), 144.05 (C_4), 130.57 (C_5), 118.84 (C_3), 113.77 (C_6), 110.71 (C_{10}), 102.06 (C_8), 43.82 (*C*H_2, {3.56 ppm}), 43.21 (*C*H_2, {3.02 ppm}), 42.85 (*C*H_2, {3.08 ppm}), 38.68 (*C*H_2, {3.77 ppm}). For atom labeling used for the NMR resonances assignment of **H** see Scheme S5.

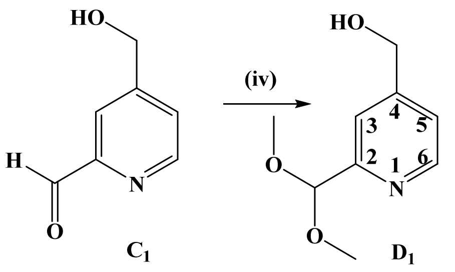

Scheme S3. Protection of aldehyde group of C_1 and atom labeling scheme used in the NMR resonances assignment of D_1.

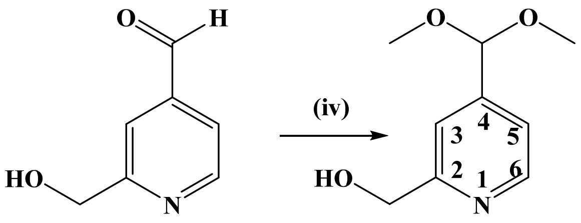

C_2 **D_2**

Scheme S4. Protection of aldehyde group of **C_2** and atom labeling scheme used in the NMR resonances assignment of **D_2**.

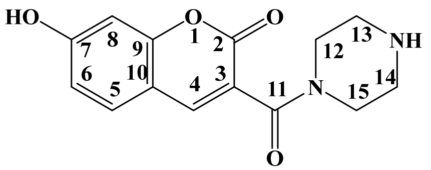

H

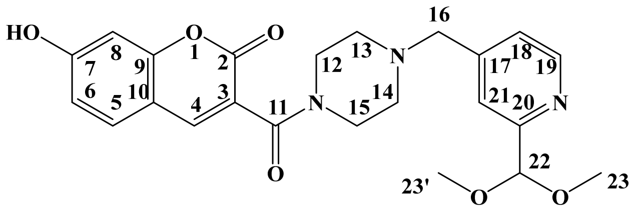

I_1

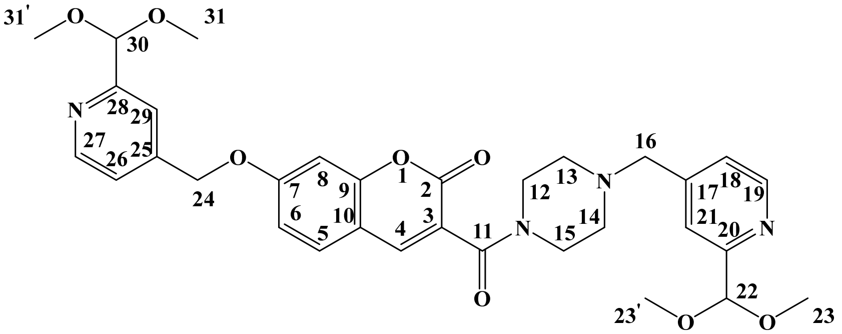

I_2

Scheme S5. Atom labeling schemes used in the NMR resonances assignment of **H, I_1** and **I_2**.

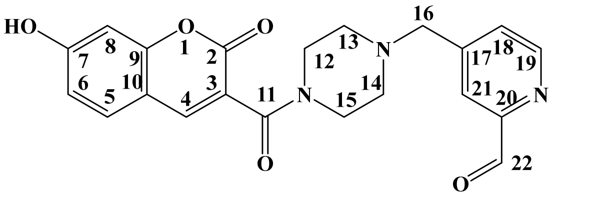

J_1

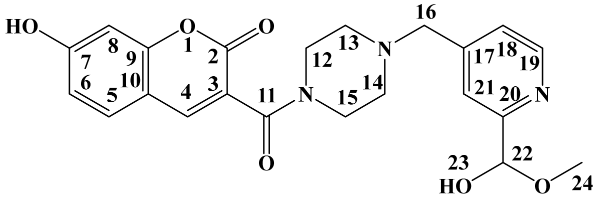

J_{h1}

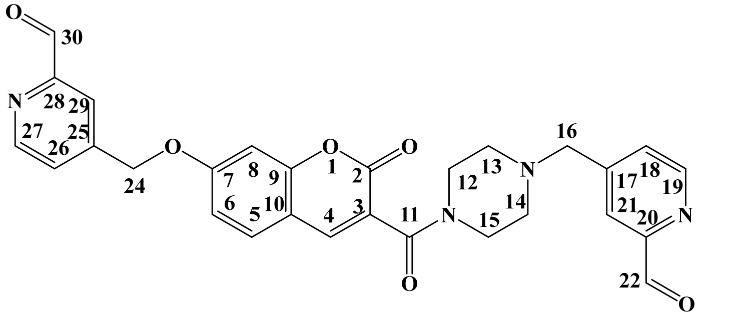

J_2

Scheme S6. Atom labeling schemes used in the NMR resonances assignment of **J_1, J_{h1}** and **J_2**.

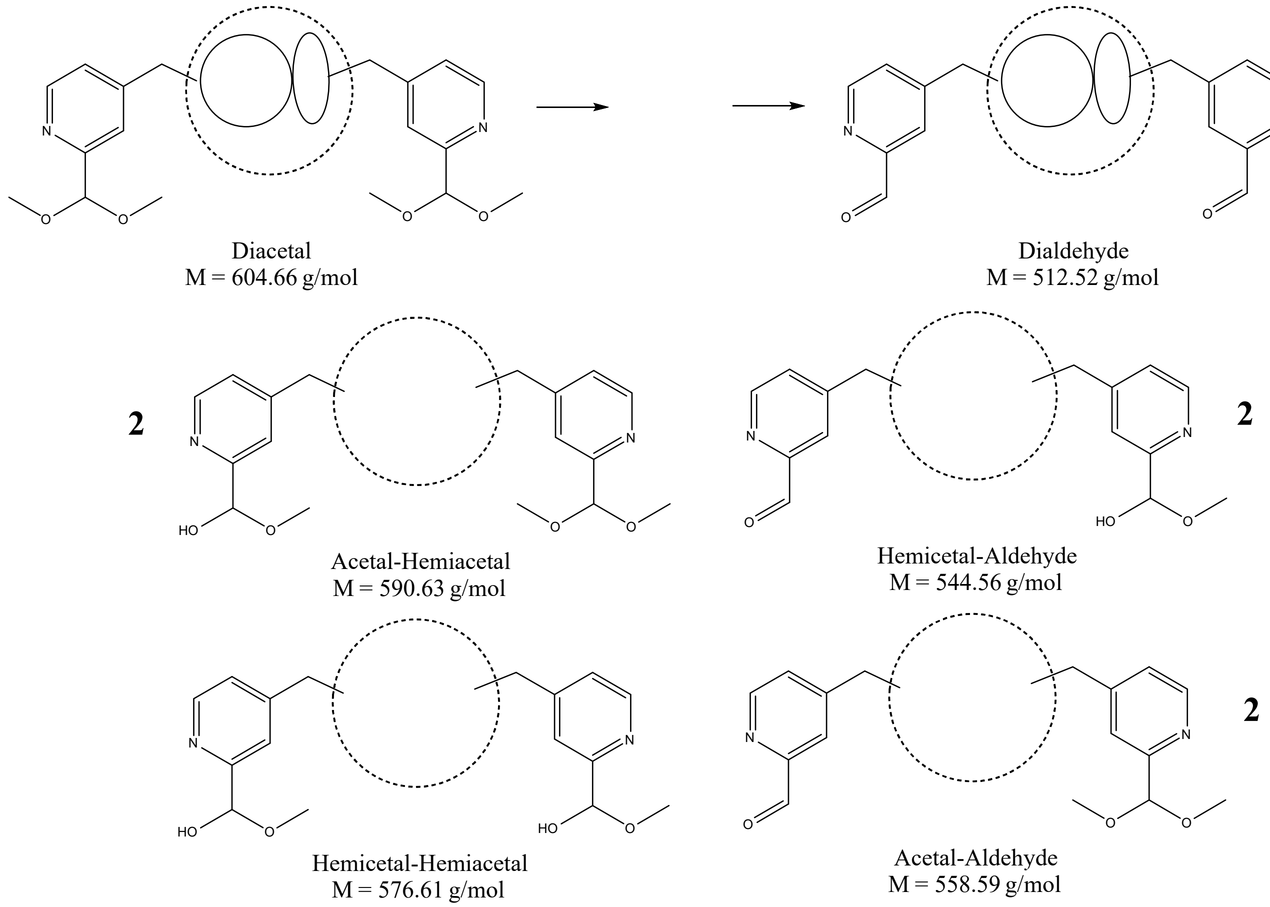

Scheme S7. Hydrolysis of two acetal groups of $\mathbf{I_2}$ in HCl solution.

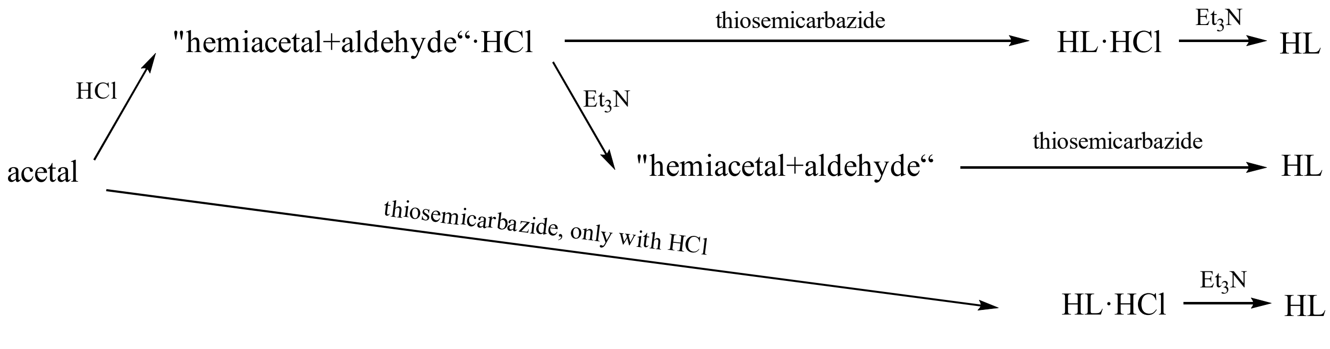

Scheme S8. Last steps in the synthesis of TSCs.

Comments to Scheme S8.

The condensation reactions of hemiacetal and aldehydes $\mathbf{J_{h1}}$, $\mathbf{J_1}$, $\mathbf{J_2}$ with 4,4-dimethyl-3-thiosemicarbazide have been investigated. Several approaches shown in Scheme S6 were explored. First, the mixture of hemiacetal and aldehyde $\mathbf{J_{h1}}$, $\mathbf{J_1}$ as hydrochlorides was allowed to react with 4,4-dimethyl-3-thiosemicarbazide in boiling ethanol for 2 h. Further work up with Et_3N (to remove HCl as $Et_3N{\cdot}HCl$) produced $\mathbf{HL^1}$ as a solid (ca 50% yield). Then the mixture of hemiacetal and aldehyde $\mathbf{J_{h1}}$, $\mathbf{J_1}$ was allowed to react with 4,4-dimethyl-3-thiosemicarbazide in boiling ethanol for 2 h to give rise to $\mathbf{HL^1}$. In both cases, no hemiacetal and aldehyde were observed after heating. These approaches demonstrated the –C=N– bond formation between hemiacetal group of $\mathbf{J_{h1}}$ as well as aldehyde group of $\mathbf{J_1}$ and amino group of 4,4-dimethyl-3-thiosemicarbazide in the presence of HCl (1) or in its absence (2). Some examples of the direct –C=N– bond formation between acetal and amino groups (in the presence of catalyst and temperatures above 100 °C in closed systems) were reported in the literature.[8,9] To examine our system the condensation of acetal $\mathbf{I_1}$ was performed in boiling ethanol in the absence and in the presence of HCl. According to ESI mass spectrometry, in this one-pot synthesis acetal $\mathbf{I_1}$ can directly react with 4,4-dimethyl-3-thiosemicarbazide under mild conditions (ethanol, 80 °C, 2 h) only in the presence of HCl, but the reaction was not complete, required longer heating as well as some additional purification procedures for $\mathbf{HL^1}$. Therefore, the synthetic procedure starting from hemiacetal and aldehyde $\mathbf{J_{h1}}$, $\mathbf{J_1}$, $\mathbf{J_2}$ (xi) was used for the synthesis of $\mathbf{HL^1}$–$\mathbf{H_2L^4}$ as more convenient (xii).

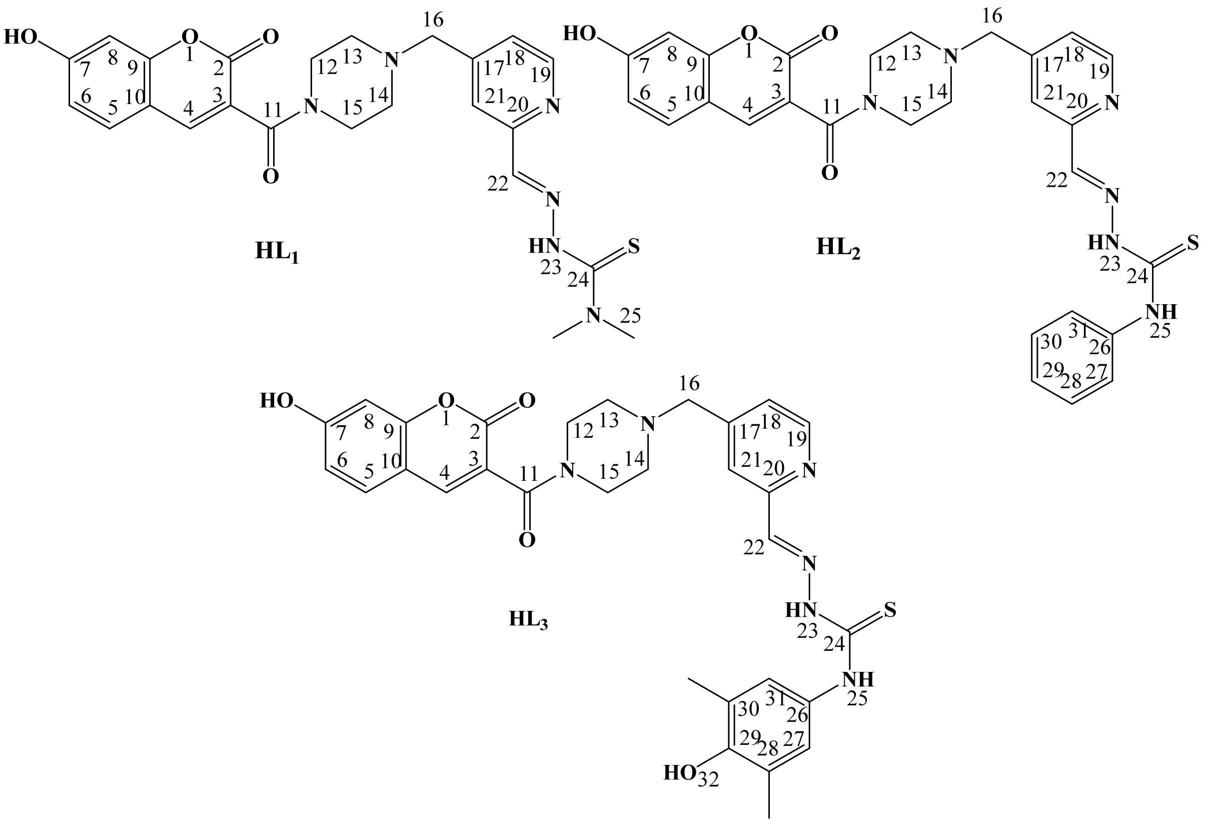

Scheme S9. Atom labeling schemes used in the NMR resonances assignment of **HL^1–HL^3**.

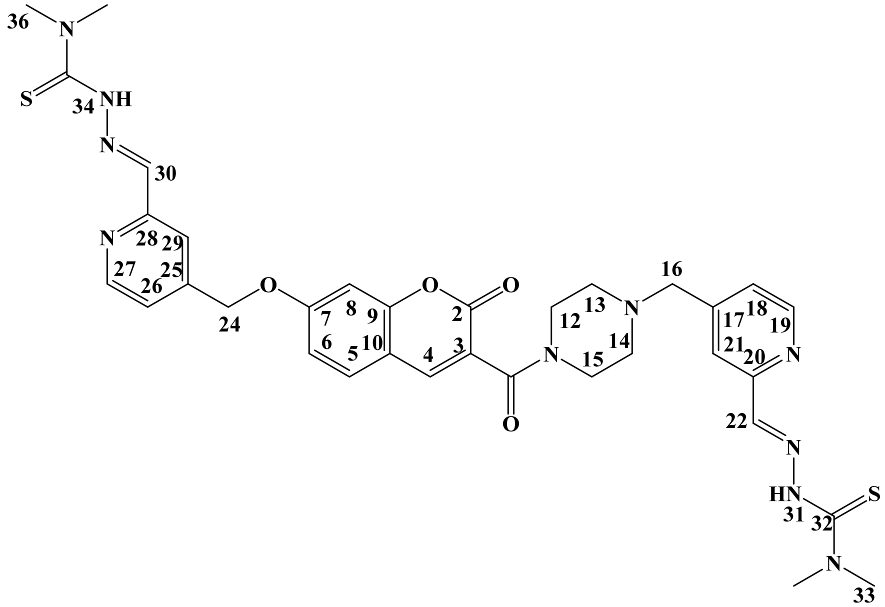

Scheme S10. Atom labeling scheme used in the NMR resonances assignment of **H$_2$L^4**.

Table S1. Optimisation of reaction conditions in step (x) to improve the yield of $\mathbf{I_1}$.

Molar ratio of pyridine/coumarin-piperazine/base E/H/base	Base	Coumarin-piperazine, H	Yield of I_1, %	Yield of I_2, %	Molar ratio I_1/I_2
**1:≤1:3**	Et_3N	H·TFA	13-18	-	-
**1:≤1:3**	TMG	H·TFA	34.2	21.9	3/1
**1:1.3:3**	TMG	H·TFA	52.2	18	5.8/1
**1:2:3**	TMG	H·TFA	67.3	traces	~30/1
**1:1.8:3**	TMG	H	79.6	11	14.5/1
**1:2:3**	TMG	H	56.9	9.5	11.9/1
**1:1.1:1.5**	K_2CO_3	H	-	43	-

References

[1] M. M. MacKeen, H. B. Kramer, K.-H. Chang, M. L. Coleman, R. J. Hopkinson, C. J. Schofield, B. M. Kessler, *Proteome Res.*, **2010**, *9*, 4082–4092.

[2] W. Chen, X. Tang, W. Dou, Z. Ju, B. Xu, W. Xu, W. Liu, *Chem. Commun.*, **2016**, *52*, 5124–5127.

[3] R. J. Warr, A. C. Willis, S. B. Wild, *Inorg. Chem.*, **2006**, *45*, 8618–8627.

[4] M. I. Dawson, R. Chan, P. D. Hobbs, W. Chao, L. J. Schiff, *J. Med. Chem.*, **1983**, *26*, 1282–1293.

[5] K. Ohui, E. Afanasenko, F. Bacher, R. Lim Xue Ting, A. Zafar, N. Blanco-Cabra, E. Torrents, O. Dömötör, N. V. May, D. Darvasiova, É. A. Enyedy, A. Popović-Bijelić, J. Reynisson, P. Rapta, M. V. Babak, G. Pastorin, V. B. Arion, *J. Med. Chem.,* **2019,** *62*, 512–530.

[6] C. Y. Ng, T. X. W. Kwok, F. C. K. Tan, C.-M. Low, Y. Lam, *Chem. Commun.*, **2017**, *53*, 1813–1816.

[7] B. Dong, X. Song, C. Wang, X. Kong, Y. Tang, W. Lin, *Anal. Chem.*, **2016**, *88*, 4085–4091.

[8] Z.-J. Li, S.-Y. Ding, H.-D. Xue, W. Cao, W. Wang, *Chem. Commun.*, **2016**, *52*, 7217–7220.

[9] L. R. Holloway, P. M. Bogie, Y. Lyon, C, Ngai, T. F. Miller, R. R. Julian, R. J. Hooley, *J. Am. Chem. Soc.*, **2018**, *140*, 8078–8081.
